# Scientific publishing: crisis, challenges, and new opportunities

**DOI:** 10.3389/fpubh.2024.1417019

**Published:** 2024-07-24

**Authors:** Paolo Vineis

**Affiliations:** School of Public Health, Imperial College London, London, United Kingdom

**Keywords:** peer review, mega-journals, quality, bias, guidelines

## Assessment of the problem

Sources that are usually considered authoritative [see for example: (1, 2)] have raised alarms about the current situation of biomedical publishing and the decline of its quality. Though it is likely that the problem affects other scientific fields, vested interests seem to be much greater in the biomedical field. So far mainly opinion pieces have been published, and a systematic bibliometric analysis would be useful. However, the perception is widespread, and the decline may produce a snowball effect, because if the quality of the primary publications (that are supposed to describe “reality” with some credibility) is poor, then also downstream systematic reviews will be poor, and hence policy decisions. For example, the best source of information on environmental carcinogens are the Monographs on the identification of carcinogenic hazards to humans of the International Agency for Research on Cancer (https://monographs.iarc.who.int/), which are widely used for policy decisions. They are authoritative because the evaluation of the evidence is well-structured, transparent and collegial. It is the scientific community to take responsibility for the credibility of the IARC Monographs. This is a good example because it is based on the principles of good methodology and lack of conflicts of interest. Within the IARC Working Groups that evaluate the evidence, it happens that some papers are discarded because of poor methodology, or the weight they are assigned in the evaluation is low for the same reason. Papers of poor quality inflate the evidence, can lead to bias in either direction (over-estimating or under-estimating risks of disease) and at the end damage the whole evaluation process and the transfer into public health.

Often it is not possible to separate weak or biased papers from the good ones. In fact, there is a gradient, from papers based on well-conducted randomized trials or on well-known collections of data (like large epidemiological prospective studies, carefully analyzed and with a sound discussion on potential bias) down to purely fabricated papers, with all the intermediate nuances. Clearly what makes the difference is the ability of the editors and reviewers to assess the methodology, which in turn depends on at least four elements: (a) the fact that there is a shared understanding of the principles of clinical, epidemiological and statistical methods; (b) that editors and reviewers are sufficiently trained and have sufficiently long experience or at least are supervised; (c) that the workload is not excessive for editors and reviewers, across journals; (d) that there are no vested interests at stake.

Looking at publishing at large, i.e., at the “*publishing ecosystem*”, all of these pre-conditions are often violated in several ways. Vested interests arise not only because authors may be supported economically (and in other ways) by industries and other entities with interests that go against public health, but also because publishers themselves are private enterprises with their own commercial goals. This requires very clear rules and transparency, for example not to multiply publications at the expense of their quality. Second, the sheer size of the scientific publishing industry today (with an exponential increase in journals and papers) (3) makes the work of reviewers extremely difficult. I receive myself approximately at least one review request per day, excluding those from predatory journals (that I reject). This means that the response rate among the reviewers who are approached is going down dramatically, i.e., that conditions (a) and (b) above will not be met easily. Reviewers have little time to apply rigorous methodological principles and editors have little time to supervise them. This is the reality that we need to acknowledge, in the interest of the scientific community and of the public. I believe we should raise these problems across all (non-predatory) journals, partly because what I described is the consequence of increasing competition.

As I have already suggested before in a commentary (4), there is the additional issue of careers. Scientific publishing is related to careers in many countries of the world, i.e., there is a strong pressure for professionals in biomedical sciences and public health to publish. This was boosted by the infodemic related to COVID-19. There is an objective conflict between the two goals, the traditional one underlying the birth itself of scientific journals (make good science broadly available), and the newer goal related to careers.

A paradox is that emerging countries tend to produce many more papers—also related to career opportunities—but not an equivalent number of reviewers. But this could be a temporary problem.

## The publishing ecosystem and its value chain

Scientific publishing should be viewed as an ecosystem, that is, a system with a large number of interactions between its components and the surrounding environment. This creates a value chain, that is, from the very beginning of scientific production added value is produced that is transferred to other downstream actors and components. The value can increase or decrease with each step. In the case of medicine, the first step is laboratory, or clinical observations, or population science (including ideas, observations, theories...), usually supported by funding, particularly public grants. Next comes publication, which can increase or decrease the value. Then come readers, media, journalists, politicians, and also academic promotions. We are observing a rapid and complex transition that, depending on the segments considered, can add or reduce the value of the chain and of the end product. What is the role of intermediation by editors and reviewers, but also the technical staff of journals (or even AI-based software)? Many editors-in-chief have been accustomed in the past to journals whose associate editors belonged to a small network of scientists (think of physics in the early 20th century: peer review was based on a small community of peers where there was little intermediation). Intermediation by a broader network of associate editors and independent reviewers then grew and became a guarantee of good quality, but it has also become a problem because of the anonymity of such networks (some journals use AI-guided algorithms to choose reviewers), leading to a low qualitative level of reviews.

There are also positive aspects to current mega-magazines: an expanded community of authors with an outreach to less developed countries; a greater variety of topics, not necessarily restricted to dominant theories; an expanded community of reviewers and editors, with a variety of cultural backgrounds; a greater possibility of influencing local policies in various ways. However, the exponential expansion of the community of authors and readers, and publishing as a business require clear rules and awareness. The goal is to maximize the added value of publishing, and also to be sure that the value does not decrease (too much) in later stages. A decoupling of careers from publishing as such, or at least criteria other than Impact Factor or H-index should be seriously discussed at all levels.

## How to redress the problem?

There are several ways to redress this problematic situation of scientific publishing (Table 1). Of course the problem is not new (though it is probably exploding) and has been addressed before for example by the COPE guidelines (Committee on Publication Ethics: https://publicationethics.org/). One way is to multiply and refine tools for the early identification of frauds, fake papers, paper mills, etc. In addition, I suggest to introduce some metrics that should be made public, like the following (part of which are contained in COPE):

- Statements on conflicts of interest of prospective authors, editors and reviewers
- Clear instructions for authors e.g., on papers out-of-scope or poor study designs (e.g., cross-sectional studies, with exceptions)
- Response rates of potential reviewers (ratio between those who accepted and those who have been approached).
- Acceptance rates and rejection rates of papers (at different phases of the process)
- Retraction rates
- Median time from submission to acceptance.

**Table 1:** Critical aspects of biomedical publishing ecosystem.

• Too many journals with overlapping scope (this decreases the overall quality). • Editors and reviewers are usually not trained and work for free (profits of publishers could be re-invested into training). • Careers should be decoupled from publishing, and better criteria for the productivity of researchers should be developed in substitution of metrics such as Impact Factor or H-index. • All the steps involved in publishing should be transparent, by making metrics such acceptance rate, retraction rate, time between submission and acceptance publicly available. • Authors should be encouraged to use guidelines such as STROBE (STrengthening the Reporting of OBservational studies in Epidemiology). • Artificial Intelligence is useful in the review process (e.g., against plagiarism or overlapping, and in checking formal requirements), but cannot substitute humans in more complex judgements (i.e., contribution to advancement of knowledge).

Of course there are more nuanced issues that cannot be addressed easily by guidelines or algorithms. For example, Frontiers in Public Health (FPH) receives regularly a flow of cross-sectional analyses based on the NHANES cohort, where one exposure and one disease are analyzed each time: clearly, these papers contribute very little to our overall knowledge and underestimate the complexities of the relationships between environmental exposures and diseases.

Some journals (like FPH) have a Research Integrity Office, that analyzes for example text overlap with published literature (allowing to identify plagiarism or redundant publication); image manipulation; papermill indicators based on AI; and sometimes authors' backgrounds are analyzed in case they have previous retractions or misconducts.

Suggestions could be given to prospective authors in addition to those contained in the journal's Guidelines for authors, for example to follow the STROBE statement (STrengthening the Reporting of OBservational studies in Epidemiology: https://www.strobe-statement.org/). Journals should also find new ways for the identification and recruitment of editors of papers and reviewers. Training of these collaborators of journals, who are in fact the main actors in scientific publishing, should be considered, though it is not simple given their large numbers. In view of the huge profits generated by scientific (not only open-access) publishers (5), part of the money devolved by institutions like Universities to open-access publishers could be used for training, in a pact of mutual interest with journals. The alternative is to compensate the work of reviewers (currently totally free-of-charge) without creating conflicts of interest. Artificial Intelligence can be extremely helpful in the review process (e.g., against plagiarism or overlapping, and in checking formal requirements), but in fact AI does not seem to be capable of detecting truly promising scientific results, separating them from more marginal contributions. This stresses even more the importance of the quality of editors.

A decoupling of careers from publishing is another needed step, though the modalities are unclear. Metrics like the Impact Factor or H-index look obsolete for many reasons [see (6) for an early critique]. The fact that citations are not a mark of quality is exemplified by the observation that a large number of citations may characterize weak or controversial papers: a clear example is a paper on Alzheimer's disease, published in 2006 and recently retracted because of the recognition of serious methodological errors; in the meantime the paper has been cited >2,300 times (7). However, alternative criteria in place of IF or H-index are not obvious.

On top of such systemic changes, a major cultural change is also required from publishers of mega-journals, that allow the practice of many overlapping journals with similar scopes. This allows capturing low-quality publications that fail acceptance by a different journal. Focusing the journal scopes would greatly help the scientific community by reducing the number of journals and contrasting a competition that currently reduces rather than increasing the overall quality.

These suggestions are obviously provisional and others could be added. The goal is to create a shared platform and a *community of practice* oriented toward transparency in publishing, in the best interest of the scientific enterprise, the readers and policy-makers.

## Author contributions

PV: Conceptualization, Methodology, Writing – original draft, Writing – review & editing.
